# EXTRACELLULAR VESICLES BIOMARKERS FOR PARKINSON’S DISEASE: A SYSTEMATIC REVIEW AND DIAGNOSTIC META-ANALYSIS

**DOI:** 10.1093/geroni/igad104.3339

**Published:** 2023-12-21

**Authors:** Hash Brown Taha, Aleks Bogoniewski

**Affiliations:** University of Southern California, Los Angeles, California, United States; University of California Los Angeles, Los Angeles, California, United States

## Abstract

Parkinson’s disease (PD) and related disorders are frequently misdiagnosed due to shared symptoms and a lack of specific biomarkers, especially in early-stages. Moreover, the prediction of progression and conversion of prodromal conditions, such as RBD, is not possible. Extracellular vesicles (EVs) are tiny, lipid-bilayer bound vesicles released by all cells into the extracellular space and bodily fluids that contain cell-state-specific messages. As such, they have become potential diagnostic tools, but inconsistencies and poor reproducibility hinder their clinical translation. This meta-analysis aimed to clarify the diagnostic ability of EVs extracted from different bodily fluids, such as cerebrospinal fluid (CSF), plasma and serum, for distinguishing patients with parkinsonian disorders from one another or from healthy controls (HCs). Following PRISMA guidelines, we analyzed 21 studies using bivariate and HSROC models, focusing mainly on 1,265 patients with PD vs. 783 HCs due to limited research on other comparisons. The results showed a moderate diagnostic ability of the test (DOR = 21.6 and AUC = 0.852), but substantial heterogeneity and publication bias. The trim-and-fill method estimated two missing studies with null or low DOR. Sub-analysis of media showed that CSF-EVs performed the best (DOR = 42.4 and AUC = 0.924), while plasma-EVs showed the lowest effectiveness. Comparison of biomarkers from general EVs vs. central nervous system (CNS)-originating EVs (n = 13) showed that the former approach is more effective with less publication bias. Though promising, the emphasis should be on standardizing the field by rigorous validation to avoid making practical clinical application a reality.

